# Effect of sulphoraphane on newborn mouse cardiomyocytes undergoing ischaemia/reperfusion injury

**DOI:** 10.1080/13880209.2019.1680705

**Published:** 2019-11-05

**Authors:** Na Peng, Luping Jin, Aizhen He, Changjin Deng, Xiaoqin Wang

**Affiliations:** Department of Cardiology, Jingmen No. 1 People’s Hospital, Jingmen, China

**Keywords:** Nrf2/HO-1, oxidative stress, apoptosis, reactive oxygen species, mitochondrial membrane potential

## Abstract

**Context:** Sulphoraphane (SFN) is an isothiocyanate, having antioxidant activity, antitumor, and therapeutic effects on cardiovascular disease.

**Objective:** This study explores the mechanisms of SFN preconditioning on ischaemia/reperfusion injury (IRI).

**Materials and methods:** Cardiomyocytes were divided into four groups as follows: control group (normoxic condition), SFN group (5 μmol/L), hypoxia/reoxygenation (H/R) group (1 h, 3 h) and SFN + H/R group. Cell viability was determined by MTT method. Levels of creatine kinase (CK), nitric oxide (NO), superoxide dismutase (SOD) and maleic dialdehyde (MDA) were determined by colorimetric method. Cell apoptosis, levels of reactive oxygen species (ROS) and mitochondrial membrane potential (MMP) were determined by flow cytometry. Levels of Bax, Bcl-2, C caspase-3, NF-E2-related factor 2 (Nrf2) and haem oxygenase-1 (HO-1) were detected by Western blot.

**Results:** H/R model inhibited cell viability, increased the levels of LDH, CK, Bax and C caspase-3, and decreased the levels of NO, Bcl-2, while the effect of H/R was partially reversed by SFN. SFN treatment reduced ROS, MDA (from 4.9 nM to 2.8 nM) production, elevated SOD level (from 39.5 U/mL to 61.7 U/mL) and improved MMP damage. Under the effect of SFN, up-regulation of nuclear Nrf2 expression and down-regulation of cytosolic Nrf2 expression were observed, which led to Nrf2 nuclear translocation and enhanced the expression of HO-1.

**Conclusion:** These results suggested that SFN had a protective effect on cardiomyocytes undergoing IRI, and its mechanism may be realized via activating the Nrf2/HO-1 pathway, thereby inhibiting apoptosis. This might provide a new approach for the treatment of ischaemic heart disease.

## Introduction

One of the main causes of disability and death in the world is ischaemic heart disease (Wang et al. 2015). The reperfusion strategy is an effective method for treating ischaemic heart disease, however, reperfusion may cause persistent tissue damage, which causes myocardial damage, cardiac dysfunction, eventually leading to arrhythmia, heart failure, and death, and such a phenomenon is known as ischaemia-reperfusion injury (IRI) (Ong et al. 2015). Therefore, improving myocardial IRI is of great importance in the treatment of ischaemic heart disease. However, to the best of our knowledge, there is no significant research progress has been made in the prevention and treatment of myocardial IRI (Li et al. 2017). The mechanism of myocardial IRI is highly complicated, and there is no clear explanation. Previous study has shown that factors such as neutrophil infiltration, apoptosis, endoplasmic reticulum (ER) stress, mitochondrial damage and reactive oxygen species production (ROS) are closely related to the occurrence and development of myocardial IRI (Wang et al. 2014a). Different types of injury factors are interrelated, thus, triggering one factor can often lead to the exacerbation of another injury factor.

Sulphoraphane (SFN) is an isothiocyanate that is found in a large number of cruciferous vegetables, especially in cabbage vegetables (Wang et al. 2016). It has been reported that SFN has a desirable cancer preventive efficacy that helps inhibit inflammatory responses and cell apoptosis and protect cardiovascular function through antioxidant activity (Forster et al. 2014; He et al. 2014; Nguyen et al. 2014). In addition, it has been reported that SFN has a potential cardio protective effect, which can protect aortic smooth muscle and cardiomyocytes from oxidative stress injury in neonatal rats (Wu et al. 2014). However, the effect and mechanism of SFN on ischaemia-reperfusion (IR) induced cardiomyocytes injury are poorly understood. Therefore, in this experiment, a hypoxia/reoxygenation injury model of cardiomyocytes was constructed to help explore the effect of SFN on cardiomyocytes in neonatal mouse that experienced IRI, and the mechanism of its action was preliminarily discussed.

## Materials and methods

### Animals and reagents

Sulphoraphane (SFN) was purchased from Sigma (St. Louis, MO). Sprague-Dawley rats aged 3 days or less were purchased from Beijing Vital River Laboratory Animal Technology Co., Ltd. (Beijing, China). The Dulbecco’s modified eagle medium (DMEM), 5-bromodeoxyuridine and foetal bovine serum (FBS) were purchased from Sigma (St. Louis, MO). The phosphate buffered saline (PBS) was purchased from GIBCO (Carlsbad, CA). Collagenase II and albumin collagen digestion liquid were purchased from Sigma (St. Louis, MO). The lactate dehydrogenase (LDH) assay kit, malondialdehyde (MDA) assay kit, superoxide dismutase (SOD) assay kit, creatine kinase (CK) assay kit and nitric oxide (NO) assay kit were purchased from Nanjing Jiancheng Bioengineering Institute (Beijing, China). The Annexin-V-FITC apoptosis detection kit, total protein extraction kit, nuclear and cytoplasmic protein extraction kit, BCA protein assay kit, and JC-1 mitochondrial membrane potential assay kit were purchased from Shanghai Beyotime Biotechnology (Beijing, China).

### Primary cardiomyocytes culture

A total of 150 Sprague–Dawley rats aged 3 days or less and weighing 7–8 g were collected and placed in an environment at 15–28 °C with 45–70% humidity. A relatively stable humidity and temperature was maintained, and enough food and water were provided to the rats. The rats were anaesthetized with 1% sodium pentobarbital (Sigma, St. Louis, MO) and their limbs were fixed on operating table and the skin was disinfected with 75% ethanol. The hearts of rats were removed and placed in phosphate buffered saline. After removing the excess atrial and aorta, the hearts were cut into fragments and placed in 0.25% trypsin with EDTA (GIBCO, Carlsbad, CA) at 4 °C overnight. After 12 h, the cardiac tissues were cultured in DMEM with 10% FBS, thermostatic water bath at 37 °C for 5 min. Then, the cardiac tissues were placed in 1.0 mg/mL collagenase II and 5 mg/mL albumin collagen digestion liquid in 37 °C for a short-time digestion until the cardiac tissues completely disappeared. The cell cluster was re-suspended in DMEM containing 10% FBS. Then, the cell suspension was transferred into a culture dish with a diameter of 100 mm and incubated at 37 °C with 5% CO_2_ for 70 min. The purification of cardiomyocytes was achieved by differential adhesion and by using 5-bromodeoxyuridine (0.1 mmol/L). The cardiomyocytes were cultured in DMEM with 10% FBS in 5% CO_2_ at 37 °C. The original culture medium was replaced with DMEM containing 10% FBS every 48 h. This study has been approved by the Jingmen No. 1 People’s Hospital ethics committee (ethical approval registration number: 32574201701109).

### Construction of hypoxia/reoxygenation myocardial cells model

Cardiomyocytes were collected and subdivided into five groups as follows: control group: cardiomyocytes were cultured in normal condition with non-hypoxic reoxygenation treatment; H3R3 group: cells were reoxygenated for 3 h after 3 h of hypoxia; H3R6 group: cells were reoxygenated 6 h after 3 h of hypoxia; H3R12 group: cells were reoxygenated 12 h after 3 h of hypoxia; H3R24 group: cells were reoxygenated 24 h after 3 h of hypoxia. Hypoxia condition was 95% N_2_, 5% CO_2_ at 37 °C, and reoxygenated condition was that 95% O_2_ and 5% CO_2_ at 37 °C for reoxygenation.

### Experimental design

The cells were divided into six groups as follows: control group (cells were maintained in normoxic condition without any treatment), H/R group (hypoxia 3 h and reoxygenate 3 h) and SFN group (0.1, 0.5, 1.0 and 5.0 μmol/L). Different concentrations of SFN were added to cardiomyocytes 1 h before implementing hypoxia.

The cells were divided into four groups as follows: control group; SFN group (5 μmol/L), H/R group (hypoxia 3 h and reoxygenate 3 h) and H/R + SFN group. Different sets of treatment methods correspond to the above experiments.

### Analysis of cell viability

The cell viability was determined by the MTT method. Cardiomyocytes were seeded in the 96-well plate with cells at 4 × 10^4^ density. After experimental treatments, cells were incubated with 10 μL (5 mg/mL) MTT solution in 5% CO_2_ at 37 °C for 4 h. The operation was performed according to the manufacturer's instructions. The OD in different groups at an absorbance of 450 nm was measured by a microplate reader (Model 680, Bio-Rad Laboratories, Inc., Hercules, CA).

### Measurement of LDH level

After experimental treatments, the cultured supernatant was used to detect the lactate dehydrogenase (LDH) release level by colorimetric method according to the LDH assay kit instructions.

### Measurement of CK, NO SOD, and MDA levels

After various experimental treatments, the cultured supernatant was used to determine the levels of CK, NO, SOD, and MDA by colorimetric method according to the manufacturer's instructions.

### Detection of apoptotic cells

Having the hypoxic reoxygenate time and the SFN concentration been determined, the apoptosis rate of cardiomyocytes was detected to determine whether the SFN could prevent apoptosis in cardiomyocytes. The Annexin-V-FITC apoptosis detection kit was used to detect cell apoptosis. The cardiomyocytes were washed one time with PBS, and then digested with 0.25% trypsin. Next, the cells were centrifuged with 1000 *g* for 5 min, and the supernatant fluid was discarded. The cells were collected and re-suspended with Annexin V-FITC combination liquid. Then, 5 μL Annexin V-FITC and 10 μL propidium iodide staining solution were added. The cells were mixed and cultured in the dark at room temperature for 15 min. The stained cells were subjected by flow cytometer (Cytomics FC-500, Beckman Coulter, Brea, CA). Apoptosis rate = (positive cell stained/total endothelial cells counted) × 100%. The procedure was performed according to the manufacturer's instructions.

### Measurement of intracellular ROS level

The cardiomyocytes were washed twice with PBS and cultured with 10 µmol/L DCFH-DA (Sigma, St. Louis, MO) in the dark at 37 °C for 20 min. The expression level of ROS was determined by flow cytometer (Cytomics FC-500). Experiments were performed according to the manufacturer's instructions.

### Measurement of mitochondrial membrane potential (MMP)

MMP detection was performed by a JC-1 mitochondrial membrane potential assay kit following the manufacturer’s protocol. The cells were washed two times with PBS and digested with 0.25% trypsin, and then suspended in PBS with 0.5 ml JC-1 at 37 °C with 5% CO_2_ for 30 min. The expression level of MMP was determined by flow cytometry (Cytomics FC-500).

### Western blot analysis

Total and nuclear proteins of cardiomyocytes lysates were respectively extracted using the related protein extraction kits. The protein concentration was quantitatively detected with the BCA protein assay kit. The samples with equal amounts of protein (20 μg) were separated by 12% sodium dodecyl sulphate polyacrylamide gel electrophoresis and then blotted onto a piece of polyvinylidene fluoride membrane (PVDF, Bio-Rad Laboratories, Inc., Hercules, CA), which was then sealed with 5% skimmed milk powder at room temperature for 1 h and cultured at 4 °C overnight with the primary antibodies as follows: anti-Bcl-2 (rabbit, 1:1000, ab59348, Abcam,  Cambridge, MA), anti-Bax (rabbit, 1:1000, ab32503, Abcam,  Cambridge, MA), anti-C caspase-3 (rabbit, 1:1500, ab2302, Abcam,  Cambridge, MA), anti-GAPDH (mouse, 1:1000, ab8245, Abcam,  Cambridge, MA), anti-Lamin B1 (rabbit, 1:1000, ab16048, Abcam,  Cambridge, MA), anti-HO-1 (mouse, 1:100, ab13248, Abcam,  Cambridge, MA) and anti-Nrf2 (rabbit, 1:100, ab137550, Abcam,  Cambridge, MA). The membrane was cultured with horseradish peroxidase anti-mouse or rabbit secondary antibodies (1:2000; sc-516102, sc-2357; Santa Cruz Biotechnology, Inc., Santa Cruz, CA) at room temperature for 2 h. The protein bands were detected with an enhanced chemiluminescence (EZ-ECL kit; Biological Industries BI,  Cromwell, CT) system (Thermo, Waltham, MA), and the grey value of the strips were analyzed and counted by ImageJ (version 5.0; Bio-Rad, Hercules, CA). The expression levels of GAPDH and Lamin B1 were used as an internal standard. The integrated optical density (IOD) of the target protein bands were compared with the IOD of interior reference and the ratio result was treated as the expression level of target gene expression.

### Statistical analysis

The SPSS 19.0 (SPSS Inc., Chicago, IL) software was used to analyse statistics data. All the measurement data in this manuscript were presented as the mean ± standard deviation ($\bar{x}\pm s$). Two-tailed Student’s *t*-test or One-way ANOVA was performed to test significance of data in different groups. A difference of *p* < 0.05 was considered as statistically significant.

## Results

### The chemical structure of SFN, immunofluorescence identification of cardiomyocytes

**Figure 1.**
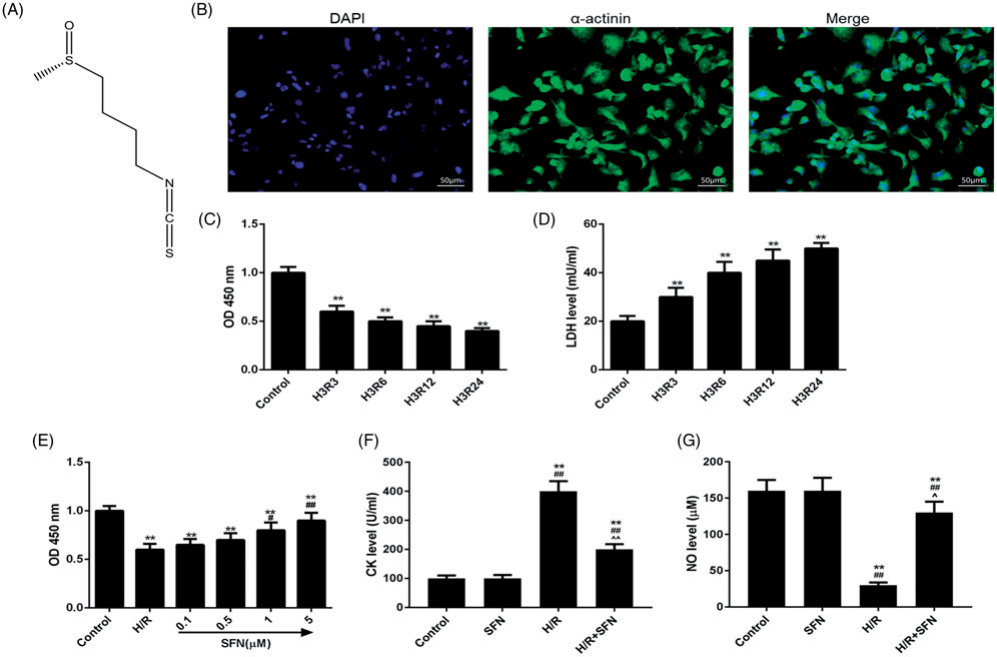
Effects of sulphoraphane (SFN) on cardiomyocytes undergoing ischaemia reperfusion injury (IRI). (A) The chemical structure of SFN. (B) Immunofluorescence identification of cardiomyocytes (bar = 200 μm). (C) The cell viability was detected by MTT colorimetric assay. (D) The expression level of lactate dehydrogenase (LDH) was determined by colorimetric. (E) The MTT colorimetric assay was used to determine the effects of different concentrations of SFN (0-5 μmol/L) on cell viability. **p < 0.01 versus control, ^#^*p* < 0.05 versus H/R, ^##^*p* < 0.01 versus H/R. (F and G) The levels of creatine kinase (CK) and nitric oxide (NO) of each group was tested by colorimetry. **p < 0.01 versus control, ^##^*p* < 0.01 versus SFN, ^^^*p* < 0.05 versus H/R, ^^^^*p* < 0.01 versus H/R.

The chemical structure of SFN is shown in Figure 1(A). Under fluorescence microscope, α-actinin specific staining was positive (green fluorescence) and DAPI positive expression of nucleus (blue fluorescence), confirmed that the cultured cells were cardiomyocytes (Figure 1(B)).

### Optimal hypoxic/reoxygenation time

The cultured cardiomyocytes were divided into five groups, and the cell viability and LDH level were tested by MTT method and colorimetric method, respectively. We found that the cell viability was significantly lower in model groups (H3R3, H3R6, H3R12 and H3R24) than that in control group after 3 h of hypoxia and 3, 6, 12 and 24 h of reoxygenation (*p* < 0.01, Figure 1(C)). The cell viability in each model group decreased significantly with prolonged duration of reoxygenation. The LDH level was significantly higher in model groups than that in control group after 3 h of hypoxia and 3, 6, 12 and 24 h of reoxygenation (*p* < 0.01, Figure 1(D)). The LDH level in each model group increased significantly with prolonged duration of reoxygenation. Thus, in order to carry out experiments with ideal cell viability and provide accurate results, 3 h of hypoxia and 3 h of reoxygenation were selected to be used in later experiments.

### The viability of cells was improved by SFN

After the hypoxia/reperfusion model was established, the cell viability in each group was determined by the MTT method. The cell viability was significantly lower in H/R group than that in control group (*p* < 0.05, Figure 1(E)). We also discovered that the cell viability increased significantly as SFN concentration increased after the cells having been pre-treated by SFN (0.1, 0.5, 1.0 and 5.0 μmol/L), particularly by 5.0 μmol/L (Figure 1(E)). Therefore, the 5 μmol/L SFN was selected for later experiments.

### SFN reduced CK level and increased NO level

After hypoxia/reperfusion experimental model and the ideal SFN concentration were determined, cardiomyocytes were divided into four groups and the levels of CK and NO in each group were tested using a colorimetric method. The results showed that CK level of H/R group was increased significantly but NO level reduced significantly, compared with the control or SFN group (*p* < 0.01, Figure 1(F,G)). In addition, CK level was significantly lower in the H/R + SFN group than that in the H/R group (*p* < 0.01, Figure 1(F)), and NO level was significantly higher in the H/R + SFN group than that in the H/R group (*p* < 0.01, Figure 1(G)).

### SFN attenuated H/R-induced apoptosis

**Figure 2.**
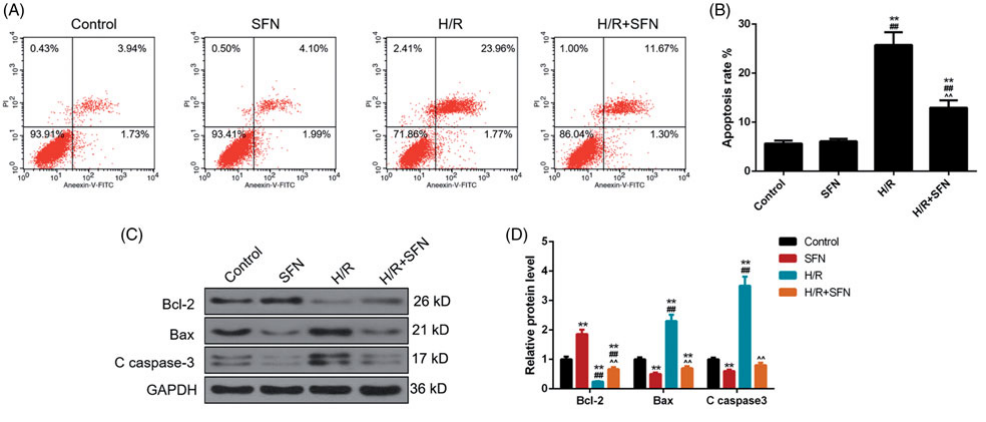
Effects of sulphoraphane (SFN) on apoptosis rate and apoptosis-related proteins of cardiomyocytes. (A) The apoptosis rate of cardiomyocytes was determined by flow cytometry. (B) Apoptosis rate in cardiomyocytes. (C) The expression of apoptosis-related protein was determined by Western blot (WB). (D) The expression levels of Bcl-2, Bax, and C caspase-3 in each group. **p < 0.01 versus control, ^##^*p* < 0.01 versus SFN, ^^^^*p* < 0.01 versus H/R.

In this study, the cell apoptosis was measured using a flow cytometer (Cytomics FC-500). The apoptosis rate was significantly higher in the H/R group than that in the control or SFN group (*p* < 0.01, Figure 2(A,B)). Compared with the H/R group, the apoptosis rate of the H/R + SFN group was decreased significantly, suggesting that SFN decreased apoptosis obviously (*p* < 0.01, Figure 2(A,B)).

### SFN improved the expression of Bcl-2 and inhibited the expression of Bax, C caspase-3

The expression level of apoptosis-related protein was tested by western blot (WB). We found that the Bcl-2 level reduced significantly after H/R treatment (*p* < 0.01, Figure 2(C,D)), while Bax and C caspase-3 levels increased significantly after H/R treatment (*p* < 0.01, Figure 2(C,D)). Compared with the H/R group, the level of Bcl-2 in the H/R + SFN group was significantly increased, while the levels of Bax and C caspase-3 were sharply reduced (*p* < 0.01, Figure 2(C,D)).

### SFN inhibited the accumulation of ROS and MDA and improved MMP and SOD levels

**Figure 3.**
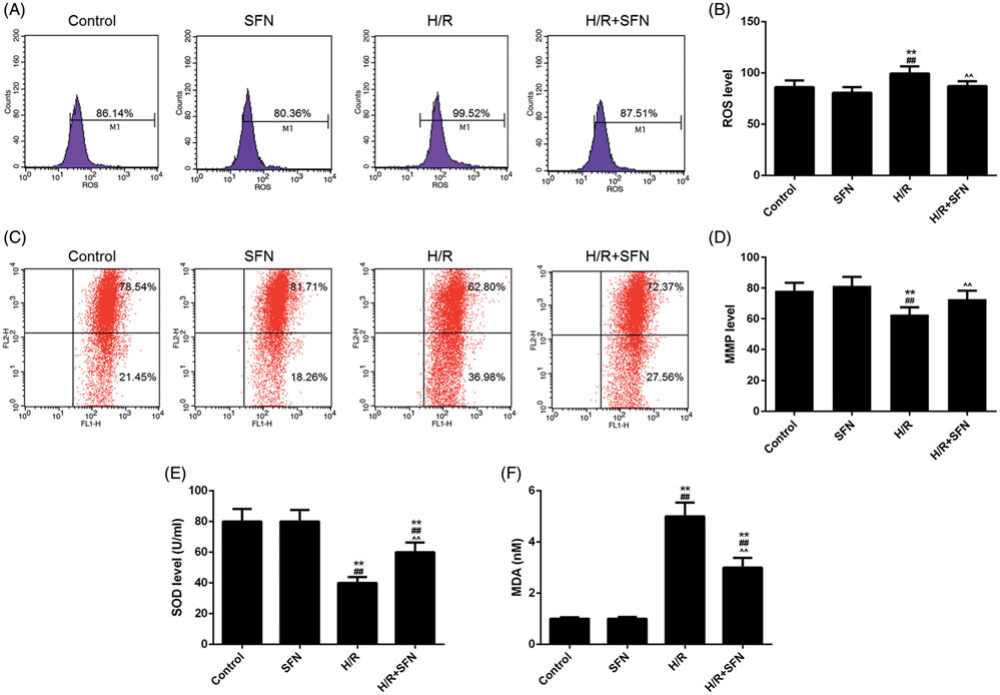
Effects of sulphoraphane (SFN) on reactive oxygen species (ROS), mitochondrial membrane potential (MMP), superoxide dismutase (SOD) and maleic dialdehyde (MDA) (A) The ROS level of cardiomyocytes of each group was determined by flow cytometry. (B) The level of ROS in the cardiomyocytes of each group. (C) The MMP level of cardiomyocytes was determined by flow cytometry. (D) The levels of MMP in the cardiomyocytes of each group. (E) The levels of SOD in the cardiomyocytes of each group. (F) The levels of MDA in the cardiomyocytes of each group. **p < 0.01 versus control, ^##^*p* < 0.01 versus SFN, ^^^^*p* < 0.01 versus H/R.

The levels of ROS and MMP were measured by flow cytometry (Cytomics FC-500), and the levels of SOD and MDA were measured by colorimetric method (Figure 3(A,C)). The ROS and MDA levels were significantly higher in H/R group than those in the control or SFN group (*p* < 0.01, Figure 3(B,F)), and the MMP and SOD levels were significantly lower in the H/R group than those in control or SFN group (*p* < 0.01, Figure 3(D,E)). Compared with the H/R group, the levels of ROS and MDA of the H/R + SFN group decreased significantly, while the levels of MMP and SOD were significantly increased (*p* < 0.01, Figure 3(B,F)).

### SFN activated NF-E2-related factor 2 (Nrf2)/haem oxygenase-1 (HO-1) pathway in cardiomyocytes

**Figure 4.**
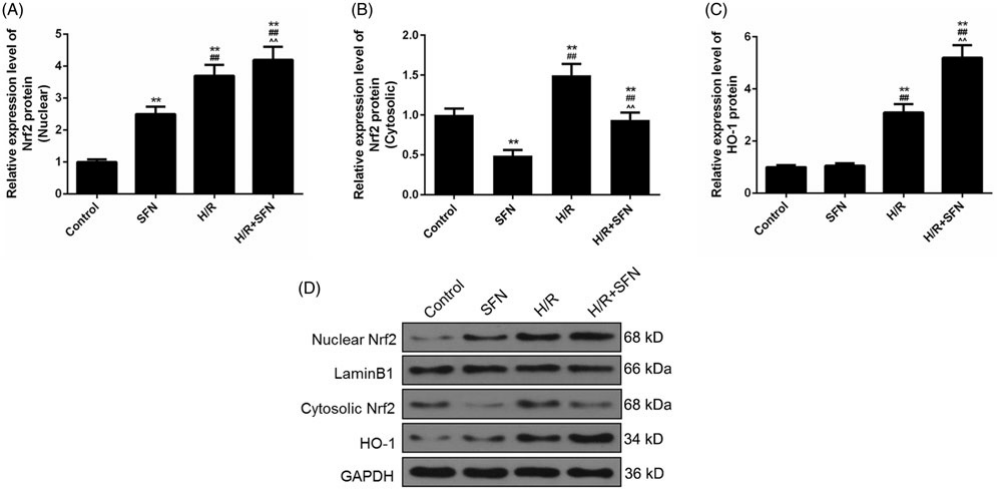
Effects of sulphoraphane (SFN) on Nrf2/HO-1 signalling pathway. (A) The level of Nrf2 in the nuclear protein of each group. (B) The level of Nrf2 in the cytosolic protein of each group. (C) The level of HO-1 protein of each group. (D) The expression of nuclear Nrf2, cytosolic Nrf2, LaminB1, HO-1 and GAPDH were detected by Western blot (WB). **p < 0.01 versus control, ^##^*p* < 0.01 versus SFN, ^^^^*p* < 0.01 versus H/R.

In this study, the effect of SFN on Nrf2/HO-1 signalling was tested by WB, and GAPDH and Lamin B1 were used as the internal standard protein. We found that the levels of nuclear Nrf2, cytosolic Nrf2 and HO-1 protein were significantly increased after H/R treatment (*p* < 0.01, Figure 4(A-D)). The levels of nuclear Nrf2 protein and HO-1 protein were significantly higher in the H/R + SFN group than those in the H/R group (*p* < 0.01, Figure 4(A,C)). The level of cytosolic Nrf2 protein was significantly lower in the H/R + SFN group than that in the H/R group (*p* < 0.01, Figure 4(B)).

## Discussion

The main pathological process of ischaemic heart disease is IRI, which has a great impact on our quality of life, health, and lives; however, there is still a lack of effective treatment (Perricone & Vander Heide 2014; Yan et al. 2015). Thus, it is highly important to find new therapeutic strategies to ameliorate reperfusion injury. SFN is an isothiocyanate extracted from cruciferous vegetables, and it has been reported that SFN has antioxidant, antitumor and anti-inflammatory properties (Corssac et al. 2018). In this study, we found that SFN could protect mouse cardiomyocytes from IRI, which was demonstrated by increased cell activity and the levels of NO, Bcl-2 and SOD, decreased CK, Bax, and C caspase-3 levels, inhibited ROS and MDA generation and improved MMP damage, activated Nrf2/HO-1 signal pathway and ultimately reduced IR-induced apoptosis.

First, the H/R experimental model and the dosage of SFN were determined. In this study, compared with the control group, the cell viability of model groups was significantly decreased, and the content of changes was more significant with the prolongation of reoxygenation time. A previous study has shown that there is a large release of LDH in cytoplasm of cardiomyocytes with IRI, and the detection of the level of LDH could reflect the degree of injury of cardiomyocytes (Li et al. 2015). We observed that the level of LDH was significantly increased after hypoxia reoxygenation treatment, and the level LDH of each model group was significantly increased with the extension of reoxygenation time. In addition, the results showed that the cell activity in the SFN group treated by different concentrations were higher than that in the H/R group (3 h of hypoxia and 3 h of reoxygenation), and the cell activity increased with the increase of SFN concentration, and 5 μmol/L SFN had the most significant effect. Therefore, to provide relatively ideal cell viability for subsequent experiments and to obtain accurate results, we determined that the optimal time to establish the H/R model was 3 h of hypoxia and 3 h of reoxygenation, and the optimal concentration of SFN was 5 μmol/L.

Studies have shown that CK and NO are important markers to detect the damage of cardiomyocytes injury (Kang et al. 2014; Xia et al. 2014). CK is one of the indicators of integrity of the cell cytoplasmic membrane and cannot penetrate the membrane under normal condition, however, when the cell membrane damage occurs, the permeability of cell membrane increases and CK penetrates from the intracellular to the extracellular matrix (Zhang et al. 2018). NO is an endogenous signal molecule, and it is considered that NO is associated with protection heart tissues and inhibition of cardiomyocytes apoptosis (Bice et al. 2016). In our study, we found that CK level of the H/R group was obviously increased, while NO level was obviously decreased, indicating that hypoxia reperfusion led to cardiomyocytes injury. Meanwhile, CK level of the H/R + SFN group was significantly decreased, while NO level was significantly increased, meaning that SFN could reduce the content of CK and increase the content of NO and protect cardiomyocytes from injury caused by hypoxia reoxygenation.

Apoptosis is the hallmark of IRI in cardiomyocytes, the Bcl-2 gene family is considered as the important regulatory genes of myocardial apoptosis, which includes pro-apoptotic proteins, anti-apoptotic proteins, sensitizer and direct activators (Chen et al. 2015). The anti-apoptosis protein Bcl-2 and pro-apoptotic protein Bax are involved in the process of IR inducing cardiomyocytes apoptosis, and Bax stimulates apoptosis, while Bcl-2 antagonises apoptosis. In addition, when cardiomyocytes were injured by IR, changes occurred to the levels of Bax and Bcl-2, leading to the release of cytochrome C from mitochondria into the cytoplasm of cells, which triggers the activation of apoptosis caspase-3, and eventually leading to cell apoptosis (Wang et al. 2014b). In this study, we found that the apoptosis rate of cardiomyocytes as well as Bax and C caspase-3 levels were significantly higher in H/R group than those in control or SFN group, whereas the Bcl-2 level in H/R group was significantly reduced, suggesting that hypoxia and reoxygenation may cause cardiomyocytes damage and lead to changes in the expression levels of Bax, C caspase-3 and Bcl-2. We also found that SFN could reduce apoptosis rate and the levels of Bax and C caspase-3 and improve the level of Bcl-2, meaning that SFN pre-treatment could improve cardiomyocytes of IRI by improving the level of Bcl-2 and reducing the level of Bax, therefore, reducing the level of C caspase-3 and the apoptosis of cardiomyocytes.

Oxidative stress is involved the process of IRI and apoptosis (Halladin 2015). Oxidative stress is characterized by the production of excessive ROS, which can lead to excessive consumption of antioxidants such as SOD and produce lipid peroxides such as MDA (Rodrigo et al. 2014; Yang et al. 2014). Meanwhile, a mass production of ROS will damage the mitochondrial membrane structure, which activates a mitochondrial permeability transition and reduces the level of MMP, finally leading to apoptosis (Yan et al. 2014). These events will result in apoptosis in cardiomyocytes. Fernandes et al. (2015) found that SFN could reduce oxidation reaction by activating PGC1-α, reducing ROS level and lipid peroxidation, increasing SOD, catalase and glutathione S-transferase activity. In our study, the results showed a significant increase in the expressions of ROS and MDA, while a decrease in the expressions of SOD and MMP after hypoxia reoxygenation treatment. Compared with the H/R group, ROS and MDA levels in the SFN + H/R group decreased significantly, and MMP and SOD levels increased significantly. These results showed that hypoxia reoxygenation treatment could induce a significant increase in ROS and MDA generation and a significant decrease in MMP and SOD levels. However, the treatment of SFN could significantly reduce the levels of ROS and MDA production and increase the levels of MMP and SOD, reduce oxidation stress and protect cardiomyocytes that experienced IRI.

Recent studies have demonstrated that nuclear factor Nrf2 is a key antioxidant enzyme inducer, which plays a highly important regulatory role in response to oxidative stress and endogenous antioxidant enzymes activation (Wang et al. 2014c; Cheng et al. 2015; Shi et al. 2016). Under normal physiological conditions, the Nrf2 is localized within the cytoplasm, while under oxidative stress, the Nrf2 is transferred from cytoplasm to nucleus where it induces the expression of the anti-oxidant HO-1 gene. Overexpression of HO-1 may protect cardiomyocytes from oxidative stress and IRI-induced apoptosis. In this study, we discovered that hypoxia reoxygenation treatment increased the levels of HO-1 protein and Nrf2 protein in cytoplasm and nucleus. Richard Zhou et al. (2014) found that SFN could activate Nrf2, increase the level of Nrf2 target haem oxygenase-1 and subsequently lowered oxidant stress. In our study, SFN could up-regulate the expression of HO-1 and nuclear Nrf2 and down-regulate cytosolic Nrf2 expression. These results suggest that as an agonist of Nrf2 protein, SFN enhanced the transfer of Nrf2 from cytoplasm to nucleus and could up-regulate the level of HO-1 protein by activating the expression of Nrf-2, therefore, attenuating the oxidative stress induced by IRI. This indicated that Nrf2/HO-1 signalling pathway participated in the protection of SFN against cardiomyocytes IRI.

In conclusion, as reported in this study, SFN preconditioning had a protective effect on cardiomyocytes experiencing IRI, and the underlying mechanism of action may be related to the activation of the Nrf2/HO-1 signalling pathway, which alleviated the apoptosis by IRI-induced. These findings provide a basis for further understanding of the application of SFN in ischaemic heart disease.

## Data Availability

The analyzed data sets generated during the study are available from the corresponding author on reasonable request.
